# *Helicobacter pylori* strains from a Nigerian cohort show divergent antibiotic resistance rates and a uniform pathogenicity profile

**DOI:** 10.1371/journal.pone.0176454

**Published:** 2017-05-02

**Authors:** Ute Harrison, Muinah A. Fowora, Abiodun T. Seriki, Eva Loell, Susanna Mueller, Margaret Ugo-Ijeh, Charles A. Onyekwere, Olufunmilayo A. Lesi, Jesse A. Otegbayo, Adegboyega Akere, Dennis A. Ndububa, Olusegun Adekanle, Ebere Anomneze, Fatimah B. Abdulkareem, Isaac A. Adeleye, Alexander Crispin, Gabriele Rieder, Wolfgang Fischer, Stella I. Smith, Rainer Haas

**Affiliations:** 1 Max von Pettenkofer-Institut für Hygiene und Medizinische Mikrobiologie, Ludwig-Maximilians-Universität, Munich, Germany; 2 Molecular Biology and Biotechnology division, Nigerian Institute of Medical Research, Yaba, Lagos, Nigeria; 3 Department of Microbiology, University of Lagos, Lagos, Nigeria; 4 Pathologisches Institut, Ludwig-Maximilians-Universität, Munich, Germany; 5 Lagos State University Teaching Hospital, Lagos, Nigeria; 6 College of Medicine University of Lagos, Lagos, Nigeria; 7 University College Hospital, Ibadan, Nigeria; 8 Department of Medicine, Obafemi Awolowo University, Ile-Ife, Nigeria; 9 Health Gate Clinics, Ojuelegba Road, Lagos, Nigeria; 10 Institut für medizinische Informationsverarbeitung, Biometrie und Epidemiologie, Munich, Germany; 11 Bavarian Health and Food Safety Authority, Oberschleißheim, Germany; 12 German Center for Infection Research (DZIF), LMU Munich, Germany; Oita University Faculty of Medicine, JAPAN

## Abstract

Antibiotic resistance in *Helicobacter pylori* is a factor preventing its successful eradication. Particularly in developing countries, resistance against commonly used antibiotics is widespread. Here, we present an epidemiological study from Nigeria with 111 isolates. We analyzed the associated disease outcome, and performed a detailed characterization of these isolated strains with respect to their antibiotic susceptibility and their virulence characteristics. Furthermore, statistical analysis was performed on microbiological data as well as patient information and the results of the gastroenterological examination. We found that the variability concerning the production of virulence factors between strains was minimal, with 96.4% of isolates being CagA-positive and 92.8% producing detectable VacA levels. In addition, high frequency of bacterial resistance was observed for metronidazole (99.1%), followed by amoxicillin (33.3%), clarithromycin (14.4%) and tetracycline (4.5%). In conclusion, this study indicated that the infection rate of *H*. *pylori* infection within the cohort in the present study was surprisingly low (36.6%). Furthermore, an average gastric pathology was observed by histological grading and bacterial isolates showed a uniform pathogenicity profile while indicating divergent antibiotic resistance rates.

## 2 Introduction

*Helicobacter pylori (H*. *pylori)* is a microaerophilic, highly motile, Gram-negative bacterium, which resides in the human gastric mucus layer. *H*. *pylori* contains and produces multiple virulence factors, including the cytotoxin-associated gene A (*cagA*) and the vacuolating cytotoxin (*vacA*), both of which are associated with a marked increase in the risk of disease development [1]. The 37 kb *cag*-pathogenicity island (*cag*-PAI) encodes *cagA*, as well as the *cag* Type IV Secretion System (*cag*-T4SS), which can form a membrane-spanning secretion channel and an extracellular pilus. Via contact with the target-cell receptor α5β1 integrin heterodimer [2], the T4SS translocates the effector protein CagA into host cells [3] and causes secretion of the proinflammatory chemokine interleukin-8 (IL-8) from gastric epithelial cells [4]. Injected CagA is tyrosine-phosphorylated on a number of Glu-Pro-Ile-Tyr-Ala (EPIYA) motifs within the C-terminal region of CagA protein, allowing it to interact with several cellular protein partners [5]. Due to these downstream effects and the correlation with cancer, CagA is considered to be a bacterial oncoprotein [6]. VacA, a pore-forming toxin, induces vacuoles in gastric epithelial cells [7], but has a number of other effects, such as inhibition of proliferation and IL-2 secretion by T cells [8] and induction of apoptosis in gastric epithelial cells [9].

Infection with *H*. *pylori* is a major cause of gastroduodenal disease, including chronic and active gastritis, peptic ulcer disease, MALT lymphoma and gastric carcinoma [10]. The prevalence within the human population is highly variable, ranging from approximately 20% of the population within industrial countries and approaching 80% in developing countries [11]. In developing countries such as Nigeria antibiotic resistance rates can reach up to 100%, a major reason for eradication failure. There is so far a lack of epidemiological and all-encompassing studies in Nigeria, with only five publications concerning the status of antibiotic resistance in Nigeria published in 1999, 2001, 2007, 2009 and 2013 [12–16]. In these studies between 31 and 186 patients were analyzed, and antibiotic resistance of *H*. *pylori* was determined to be as follows: amoxicillin (0% - 100%), clarithromycin (12.7% - 100%), metronidazole (40% - 100%), and tetracycline (11% - 100%). Thus, our knowledge about the current state of drug resistance is low and, due to small sample sizes, the data show wide variation. To better understand the status and consequences of *H*. *pylori* infections in Nigeria, we studied the antibiotic resistance of isolated strains and attempted to correlate this to patient data, demographic patterns, disease outcome and characteristics of the isolated strains.

To accomplish this, we established a network of eight hospitals in Nigeria, with coordination centers located in Lagos [17] and Munich (Germany). To study the epidemiological situation, this network was used to collect biopsies, patient-isolated strains, patient questionnaires and the according diagnosis of the gastroenterologist, which was finally statistically correlated. This provided a very detailed characterization of the isolates, which included investigation of antibiotic resistance, and placed it in context with the patient information. From this work, involving 577 patients, a clearer overview of the current situation of *H*. *pylori* infections in Nigeria was obtained.

## 3 Methods

### 3.1 Study design

577 patients were recruited between August 2010 and December 2013 in eight different hospitals in Nigeria. Five hospitals were located in Lagos, one in Ibadan, one in Ile-Ife and one in Jos. A questionnaire, a form with gastroenterological results and a signed consent were filled out by and with each patient, respectively. Furthermore an urease breath test (UBT), the gold standard in *H*. *pylori* diagnostics, was performed, and six biopsies (three from the antrum and three from the corpus) were taken from each patient. One biopsy from the antrum was used to confirm the *H*. *pylori* infection by a *campylobacter*-like organism (CLO) test, one antrum and one corpus biopsy were used for isolation of the *H*. *pylori* strain responsible, while the other three biopsies were used for histological analysis.

Inclusion criteria were the treatment in one of the eight mentioned hospitals, the agreement to participate this study, and being a patient showing both stomach afflictions and an expected *H*. *pylori* infection. Furthermore, patients with prior antibiotic treatment were not excluded (see discussion).

**Fig 1:**
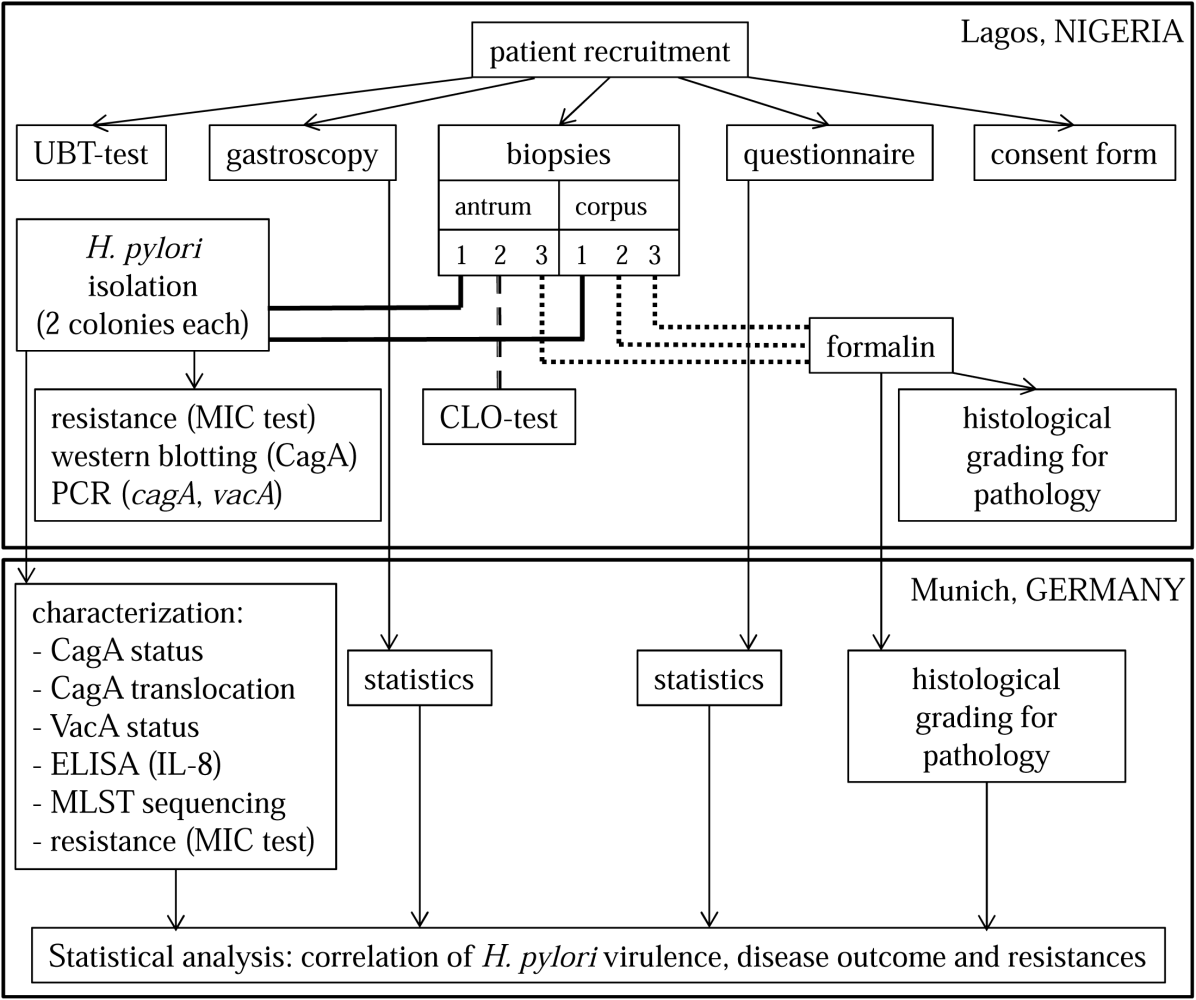
Study design.

The recruitment, the gastroenterological examination, the UBT, the CLO test, the fixation of the biopsies, the isolation of the strains, MIC test, polymerase chain reaction (PCR), western blotting as well as the histological grading were performed in Nigeria (Fig 1). Throughout the course of this study, 577 patient questionnaires, 571 forms with gastroenterological results, 351 fixed biopsies (from 136 patients) and 111 isolates (from 50 patients) were sent to Munich (Germany). Due to the challenging field conditions in Nigeria, 111 isolates is the final number and all following results refer to this number. In Munich, further characterization of the isolates, the repetition of the MIC test, the western blotting, the PCR, the procedure of the enzyme linked immunosorbent assay (ELISA), the confirmation of the histological grading as well as the final statistical analysis were performed–thus determining any correlation between *H*. *pylori* virulence, antibiotic resistance, patient information and disease outcome.

### 3.2 Urease breath test

The urease breath test (UBT) test was obtained from Tri-Med Distributors in Australia. Potentially-infected patients fasted either overnight or for a minimum of four hours. A PYtest capsule containing a known amount of ^14^C-labelled urea was provided to patients to drink with 30 ml of water, followed after 3 minutes by a further 30 ml of water. After further 7 min a breath sample was collected in a sterilized mylar balloon. Following contact between ^14^C-labelled urea and stomach-resident *H*. *pylori*, the molecule was hydrolyzed into ^14^C-carbon dioxide and ammonia. The carbon dioxide then entered the bloodstream and was in turn exhaled by the patient. The collection balloon containing patient breath samples was then analyzed either directly in the hospital or sent to a pathology laboratory for analysis. The contents of the balloon were dissolved into breath collection fluid and then liquid scintillation fluid was added to quantify the degree of ^14^C present. Scintillation values below 50 DPM (disintegrations per minute) were considered to be negative, values over 200 DPM as positive and all values between as “borderline”.

### 3.3 CLO test

The *campylobacter*-like organism (CLO) test was obtained from Tri-Med Distributors in Australia. The test kit contains urea, and utilizes a pH-mediated color change from yellow to red as a marker for the presence of *H*. *pylori* within the biopsy.

### 3.4 *H*. *pylori* isolation

The biopsy sample was rolled several times over a GC agar serum plate (Oxoid) containing horse serum (80 ml/l), IsoVitaleX^TM^ (10 ml/l; from BD, Germany), vancomycin (10 mg/l), trimethoprim (5mg/l), and nystatin (1 mg/l). Plates were then incubated for 5 to 9 days in a microaerobic atmosphere (85% N_2_, 10% CO_2_, 5% O_2_) at 37°C. Isolated bacteria were passaged twice to obtain a vital and dense culture before storage at -80°C. Since it is known that a patient can harbor more than one strain, we have analyzed up to two colonies per antrum and corpus, respectively.

### 3.5 Transport of strains

For transport of the isolates from Lagos to Munich (Germany), strains were cultured on GC agar serum plates (see above), passaged twice and transferred into Portagerm Pylori (bioMérieux SA Mercy L’Etoile France) medium. In this state they were shipped to Munich (via DHL couriers).

### 3.6 PCR

PCR was performed in 25μl reaction mixtures, consisting of x1 PCR buffer, magnesium chloride (1.5 mM), dNTP (200 μM), primer (20 pmol) and 1U Taq DNA polymerase (Pan-biotech). Amplification was carried out in a Peqlab Thermocycler using the following cycling parameters: initial denaturation at 95°C for 5 min, followed by 35 cycles of 95°C for 30 sec, 54°C for 30 sec and 72°C according to amplicon length (1 min per 1000 bp). This was followed by a final extension of 72°C for 10 min. The *hpWAfrica* strain J99 [18] and the *hpEurope* strain P12 [19] were used as control strains. Primers are listed in Table 1.

**Table 1:** Primer sequences.

Target gene	Sense primer	Antisense primer	Amplicon length
*16S rRNA*	AAGGCTATGACGGGTATCCG	GGGGGTTGCGCTCGTTGCGGG	826 bp
*23S rRNA*	CCACAGCGATGTGGTCTCAG	GGGACCGAACTGTCTCACGACG	878 bp
*cagA*	CCATCGATGGTAAAAATGTGAATCGT	CAGGTACCGCGGCCGCTTAAGATTTTTGGAAACCAC	3700 bp
*cagA*	ACCGCTCGAGAACCCTAGTCGGTAATGGG	ATATCGATTTAAGCCAATTTTTGATTCCTTG	500 bp
*dupA*	CTACAATATAGCTCTCAAAAG	AGCAATAAAACGCTTAAAAGTCTC	2959 bp
*dupA*	GCCAGAGATTTCAATGATGTC	AAAAATTTAGGCTCAAAGTCTG	970 bp
*vacA s1*	CTGCTTGAATGCGCCAAAC	ATGGAAATACAACAAACACAC	259 bp
*vacA s2*	CTGCTTGAATGCGCCAAAC	ATGGAAATACAACAAACACAC	286 bp
*vacA m1*	GGTCAAAATGCGGTCATGG	CCATTGGTACCTGTAGAAAC	290 bp
*vacA m2*	CATAACTAGCGCCTTGCAC	CATAACTAGCGCCTTGCAC	352 bp
*atpA*	GGACTAGCGTTAAACGCACG	CTTGAAACCGACAAGCCCAC	841 bp
*efp*	GGCAATTTGGATGAGCGAGCTC	CTTCACCTTTTCAAGATACTC	559 bp
*mutY*	GTGGTTGTAGYTGGAAACTTTACAC	CTTAAGCGTGTGTYTTTCTAGG	676 bp
*ppa*	GGAGATTGCAATGAATTTAGA	GTGGGGTTAARATCGTTAAATTG	706 bp
*HptrpC*	TAGAATGCAAAAAAGCATCGCCCTC	TAAGCCCGCACACTTTATTTTCGCC	633 bp
*ureI*	AGGTTATTCGTAAGGTGCG	GTTTAAATCCCTTAGATTGCC	686 bp
*yphC*	CACGCCTATTTTTTTGACTAAAAAC	CATTYACCCTCCCAATGATGC	734 bp

### 3.7 Western blotting

Rabbit polyclonal antisera against CagA and VacA have been described previously [20]. Sodium dodecyl sulfate-polyacrylamide gel electrophoresis (SDS-PAGE) and Western blotting was performed as described [4], using polyvinylidene difluoride (PVDF) filters blocked with 5% non-fat milk powder in TBS (50 mM Tris-HCl, pH 7.5, 150 mM NaCl), 0.1% (v/v) Tween 20. Alkaline phosphatase-conjugated protein A or horseradish peroxidase-conjugated anti-rabbit IgG antiserum was used to visualize bound antibody. The *hspWAfrica* strain J99 [18] and the *hpEurope* strain P12 [19] were used as control strains.

### 3.8 Phosphorylation of translocated CagA

Standard infections of AGS cells with *H*. *pylori* strains and subsequent preparations for phosphotyrosine immunoblotting were performed as described previously [3]. Briefly, cells were infected with bacteria at a multiplicity of infection of 100 for 4 h at 37°C, washed three times and suspended in PBS containing 1mM EDTA, 1mM Na_3_VO_4_, 1mM PMSF, 10μg/ml leupeptin, and 10μg/ml pepstatin. Cells with adherent bacteria were collected by centrifugation and resuspended in sample buffer. Tyrosine-phosphorylated proteins were analyzed by immunoblotting with the phosphotyrosine antiserum PY99 (Santa Cruz Biotechnologies). The *hspWAfrica* strain J99 [18] and the *hpEurope* strain P12 [19] were used as control strains.

### 3.9 IL-8 secretion by ELISA

The production of IL-8 by AGS cells after infection with *H*. *pylori* strains for 4 h was determined from cell supernatants by a sandwich enzyme linked immunosorbent assay (ELISA), as previously described [4]. The *hpEurope* strain P12 [19] was used as control strain.

### 3.10 MLST

For strain phylogeny, we used multilocus sequence typing analysis [21]. Partial nucleotide sequences of the housekeeping genes *atpA*, *efp*, *mutY*, *ppa*, *trpC*, *ureI* and *yphC* were determined for each strain by sequencing the corresponding PCR products; primer pairs are listed in Table 1. The resulting sequences were concatenated and aligned with the corresponding sequences from 345 reference strains from the MLST database [21] and from 45 fully sequenced genomes [22], using the Muscle algorithm within MEGA5.2 [23]. Phylogenetic trees were constructed and tested by neighbor joining with MEGA5.2, using the Kimura 2-parameter model of nucleotide substitution, and 1,000 bootstrap replications.

### 3.11 MIC test

The minimal inhibitory concentration (MIC) test was obtained from Bestbion in Germany. The strains were grown as a liquid culture in Brucella Broth + 10% fetal calf serum (FCS) overnight until OD 1.0. 400 μl of the liquid culture was transferred to a GC agar serum plate (see above), after which an appropriate antibiotic strip was placed on the plate. Antimicrobial agent concentrations ranged from 0.016 to 256 μg/mL. After two days incubation the zone of inhibition was measured according to the manufacturer’s instructions under consideration of recommended MIC breakpoints (EUCAST Clinical Breakpoint Table v. 6.0, valid from 2016-01-01): amoxicillin 0.125–0.125 μg/mL, clarithromycin 0.25–0.5 μg/mL, tetracycline 1–1 μg/mL, metronidazole 8–8 μg/mL (range describes the value between sensitivity ≤ and resistance >). The *H*. *pylori* strains P12 [19] and J99 [18] were used as control strains.

### 3.12 Transformation

PCR products of 23S rRNA of resistant isolates were generated and transformed in *H*. *pylori* strain J99 by natural transformation, as described previously [24]. *H*. *pylori* transformants were selected on serum agar plates containing 6 mg/l clarithromycin.

### 3.13 Histology

Hematoxylin and eosin stained longitudinal paraffin sections of antrum and corpus were on the intensity of inflammation, metaplasia, and presence of gastric mucosa associated lymphoid tissue (MALT), as well as the presence and occurrence of atrophies, metaplasia, dysplasia, cancer, erosion and ulcers. Grading was performed according to the updated Sydney System [25] under blinded conditions by expert pathologists in Lagos (FBA) as well as in Munich (SM).

### 3.14 Ethics

The study was reviewed and approved by the Ethics Committee of the Nigerian Institute of Medical Research (registration number IORG0002656) and of the Ludwig-Maximilians-University Munich (registration number 335–08). The study was conducted in line with the Declaration of Helsinki and informed consent was obtained from all study participants.

### 3.15 Statistical methods

All variables were described using appropriate measures of location and dispersion, stratified for gender. Interrelations between clinical, pathologic, and laboratory findings were investigated bivariately using chi-squared tests for nominal and rank sum tests for ordinal or quantitative outcomes. Due to the exploratory character of these analyses, all tests were performed on a local alpha level of 5% without any correction for multiple testing. All analyses relied on the Statistical Analysis System SAS, version 9.2 for Linux (SAS Institute, Cary, NC).

## 4 Results

### 4.1 Low rate of *H*. *pylori* infection in Southern Nigeria

In order to prevent pre-selection of the cohort, inclusion criteria for the present study were broad, comprising patients with both stomach afflictions and a possible or expected *H*. *pylori* infection. The success of our criteria was supported by the demographic results (Table 2). The gender of the participants was balanced, 54.9% of the participants were female and the majority of patients were between 40 and 60 years old, suggesting that this group comprises a representative cross-section.

**Table 2:** Demographic data of participating patients.

		all patients (n = 577)		UBT-positive patients (n = 206)		patients with successfully isolated strains (n = 50)		infected patients: UBT-positive and/or isolated strain (n = 211)	
Sex									
	female	315	54.6%	118	57.3%	24	48.0%	121	57.3%
	male	261	45.2%		88	42.7%		26	52.0%		90	42.7%
	unknown	1	0.2%	0	0.0%	0	0.0%	211	0.0%
Age									
	0–10	1	0.2%	0	0.0%	0	0.0%	0	0.0%
	11–20	12	2.1%	4	1.9%	2	4.0%	4	1.9%
	21–30	60	10.4%	15	7.3%	4	8.0%	15	7.1%
	31–40	94	16.3%	36	17.5%	7	14.0%	36	17.1%
	41–50	125	21.7%	45	21.8%	13	26.0%	46	21.8%
	51–60	130	22.5%	50	24.3%	13	26.0%	54	25.6%
	61–70	93	16.1%	30	14.6%	4	8.0%	30	14.2%
	71–80	32	5.5%	13	6.3%	2	4.0%	13	6.2%
	81–90	8	1.4%	4	1.9%	1	2.0%	4	1.9%
	91–100	1	0.2%	0	0.0%	0	0.0%	0	0.0%
	101–110	2	0.3%	0	0.0%	0	0.0%	0	0.0%
	unknown	19	3.3%	9	4.4%	4	8.0%	9	4.3%
UBT test									
	positive	206	35.7%	206	100.0%	45	90.0%	206	97.6%
	negative	276	47.8%	0	0.0%	4	8.0%	4	1.9%
	borderline	74	12.8%	0	0.0%	1	2.0%	1	0.5%
	not recorded	21	3.6%	0	0.0%	0	0.0%	0	0.0%
City of examination									
	Lagos	327	56.7%	113	54.9%	39	78.0%	117	55.5%
	Ife-Ife	104	18.0%	37	18.0%	6	12.0%	38	18.0%
	Ibadan	61	10.6%	28	13.6%	2	4.0%	28	13.3.%
	Jos	85	14.7%	28	13.6%	0	0.0%	28	13.3.%

In order to analyze the rate of *H*. *pylori* infection in the patient cohort, the results of the UBT as well as the successful isolation of *H*. *pylori* were considered. Of 577 recruited patients 206 (35.7%) showed a positive result in the UBT. Interestingly, only 74.7% of the UBT positive patients were confirmed in the CLO test (data not shown). Biopsy material from all 577 patients was used for *H*. *pylori* culture, independent of the UBT result (positive, negative or borderline) and a total number of 111 *H*. *pylori* isolates were successfully cultivated from 50 patients. Interestingly, five *H*. *pylori* isolates were successfully obtained from four UBT-negative patients and one UBT-borderline patient. A patient was defined as infected when at least one positive result was obtained from either UBT or *H*. *pylori* culture, providing a calculated infection rate of 36.6%. This is not an infection prevalence since healthy people were not included and thus, this number does not reflect the infection rate within the average Nigerian population.

### 4.2 Divergence in antibiotic resistance rates of *H*. *pylori*

**Fig 2:**
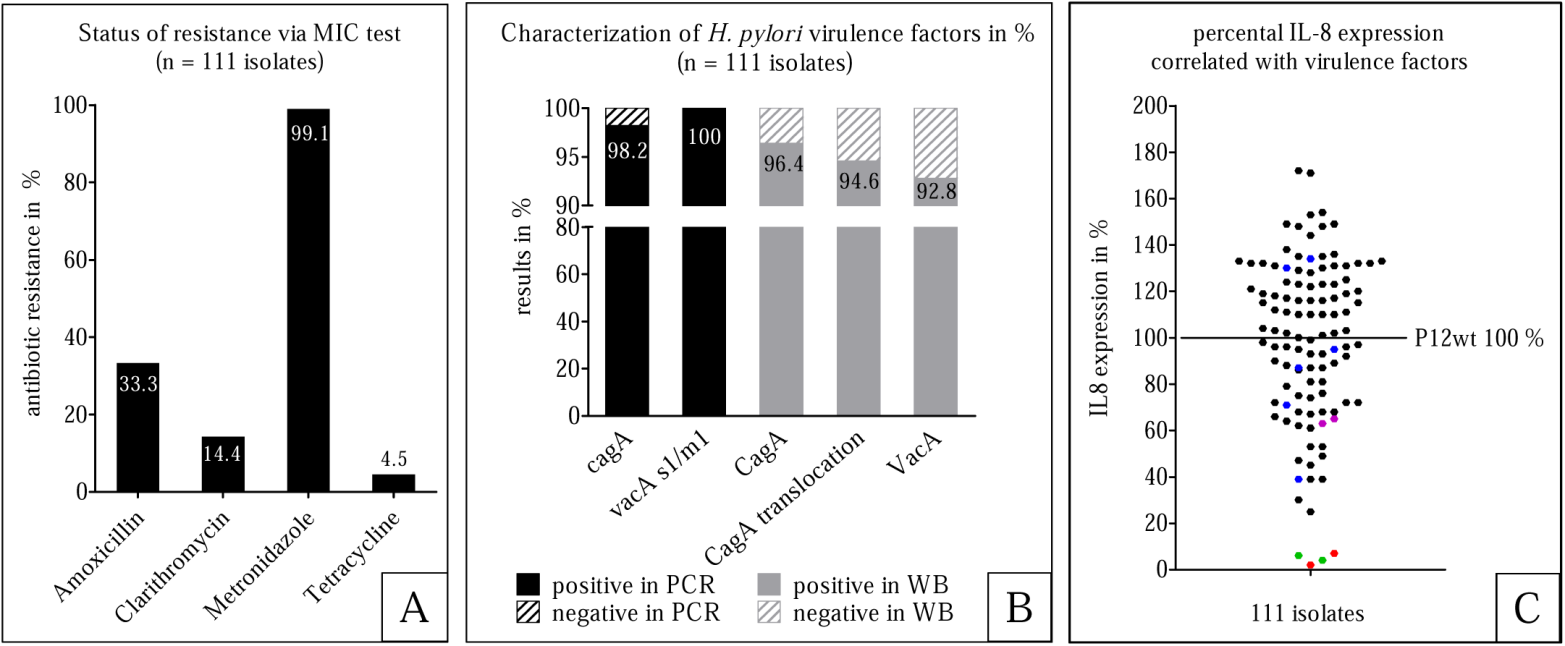
Characterization of *H*. *pylori* isolates. 111 isolated strains were characterized by the status of resistance, the analysis of the major virulence factors as well as their IL-8 expression. A: The bacterial resistance to amoxicillin, clarithromycin, metronidazole and tetracycline is shown as percentage. These results are based on MIC tests. B. Black: shows PCR results of the genes *cagA* and *vacA*. Grey: shows the Western blotting results of the proteins CagA, translocation of CagA into AGS cells, and VacA. C. Induction of IL-8 secretion by AGS cells in relation to *H*. *pylori* P12. Each dot illustrates one isolate. Black: isolates produce VacA, CagA, and are also able to translocate CagA. Green: isolates which produce neither VacA nor CagA and show no CagA translocation. Red: isolates which do produce VacA, but not CagA. Blue: isolates which produce and translocate CagA, but do not produce VacA. Purple: isolates which produce VacA and CagA, but are not able to translocate CagA.

The current rate of antibiotic resistance in Nigeria has not been decisively determined, and as high levels of drug resistance of *H*. *pylori* may play an important role in therapeutic failure, we decided to analyze the antibiotic resistance of the isolated strains. This was accomplished via MIC testing of all 111 isolates, which indicated a nearly complete bacterial resistance to metronidazole (99.1%), but lower rates of resistance to amoxicillin (33.3%), clarithromycin (14.4%) and tetracycline (4.5%) (Fig 2A). However, considering the antibiotic resistance rates separately, this study has confirmed the extremely high resistance rate of *H*. *pylori* against metronidazole in Nigeria–which is in contrast to resistance levels against tetracycline, which were surprisingly low.

We next sequenced the mid region of 23S rRNA of those 16 isolates which had shown a clarithromycin resistance in the MIC test to determine if this drug resistance was due to point mutations reported previously. Of these potential mutations, our isolates contained A2143C (2 of 16), A2143G (1 of 16) and A2144G (2 of 16), as well as other point mutations, one of which (C2196T) has been described before [26], but with an unclear role. To examine whether these mutations are involved in clarithromycin resistance, we transformed the clarithromycin-sensitive J99wt strain with 23S rRNA PCR amplification products of all 16 resistant Nigerian isolates. PCR products from resistant strains with point mutations at positions 2143 or 2144 induced clarithromycin resistance in J99wt, whereas none of the PCR products from the 11 resistant strains with other point mutations (including C2196T) induced resistance (data not shown). Thus, these other point mutations are unlikely to be responsible for the observed clarithromycin resistance. In conclusion, it is more likely that these 11 isolates utilize other mutations or a completely different mechanism of resistance encoded outside the 23S rRNA.

### 4.3 *H*. *pylori*-infected patients show an average pathology and an almost uniform presence of the major bacterial virulence factors

To analyze the disease outcome and its relation to the characteristics of *H*. *pylori* isolates, histological grading of stomach biopsies was performed. A maximum of three biopsies was obtained from each patient for histological grading (one from antrum and two from corpus); in total 351 biopsies from 136 patients were analyzed. Chronic and active inflammation was found in most of the patients stomachs, with only 30.2% of patients demonstrating the presence of MALT (lymphoid aggregates or lymph follicles). In 7.1% and 5.4% of the biopsies, atrophy or metaplasia could be observed, respectively, while erosion was discovered in only two cases. However, neither dysplasia, nor ulcer or cancer could be observed in the histology (Table 3). A statistical correlation was found between UBT-positive patients and active as well as chronic inflammation (according to histological evaluation). The inflammation in the patients was graded using a score from 0 (no inflammation) to 3 (strong inflammation). From all UBT-positive patients an average score of 1.5 was observed in active inflammation, and a score of 1.9 in chronic inflammation. In comparison, UBT-negative patients exhibited a score of 0.7 in active inflammation (p < 0.001) and a score of 1.4 in chronic inflammation (p < 0.001). These differences were statistically significant. All other potential correlations were not significant or not analyzable.

**Table 3:** Pathology analyzed by a histological grading (based on the updated Sydney System (22)) in HE stained slides.

stomach biopsies n = 351 (136 patients)							
Chronic inflammation				Metaplasia			
	0	12	(3.4%)		none	332	(94.6%)
	1	83	(23.6%)		complete intestinale m.	12	(3.4%)
	2	218	(62.1%)		incomplete intestinale m.	3	(0.9%)
	3	38	(10.8%)		compl. & incomplete m.	4	(1.1%)
Active inflammation				Dysplasia			
	0	64	(18.2%)		no	351	(100.0%)
	1	120	(34.2%)		yes	0	(0.0%)
	2	145	(41.3%)				
	3	22	(6.3%)	Erosion			
					no	349	(99.4%)
MALT					yes	2	(0.6%)
	none	245	(69.8%)				
	lymphoid aggregates	59	(16.8%)	Ulcer			
	lymphollicles	24	(6.8%)		no	351	(100.0%)
	lymphoid agg. & lymphollicles	23	(6.6%)		yes	0	(0.0%)
				Cancer			
Atrophy					no	351	(100.0%)
	none	326	(92.9%)		yes	0	(0.0%)
	focal	13	(3.7%)				
	diffuse	6	(1.7%)				
	moderate	6	(1.7%)				

As compared to histological findings, the gastroenterological findings resulted in a more serious disease outcome: 37.4% of the patients showed a normal mucosa, 32.7% erosions, 24.2% hyperemia, 15.5% ulcers and two patients revealed gastric cancer (data not shown); all other endoscopic findings (oedematous, polyps > 1cm, mucosal atrophy, haemorrhages, excrescence, hypoemia) were below 10.0% and in the order as listed. Due to multiple observations within the same patient, as well as some missing information, these percentages do not add up to 100%. Thus, the disease outcome analyzed by histology is relatively mild, but regarding the endoscopic findings the pathology is considered as average for an African country.

To determine a possible correlation between the presence of the major virulence factors and disease outcome, we examined the expression status and the functional characteristics of the *cag*-PAI and VacA of all 111 *H*. *pylori* isolates. The majority of isolates were *cagA*- (98.2%) as well as *vacA*-positive (100%), and all *vacA* sequences showed the s1/m1 genotype. *In vitro* infection experiments of AGS gastric epithelial cells were performed and showed that 105 of the 107 CagA-positive isolates were able to translocate CagA, suggesting that these isolates were also able to inject CagA in the stomach *in vivo*. Furthermore, more than half of the strains (53.2%) induced higher IL-8 expression levels in AGS cells than a commonly used laboratory strain (P12). The characterization of the virulence factors and induced IL-8 secretion levels associated with these isolates are summarized in Fig 2C and 2D).

**Fig 3:**
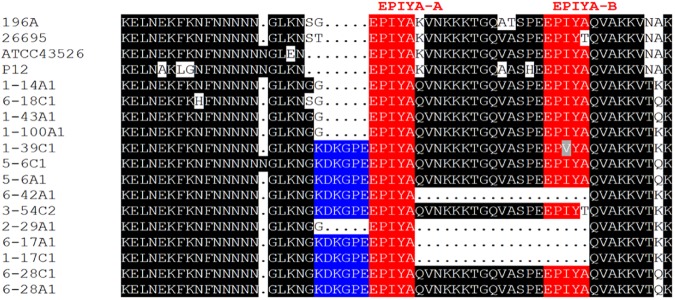
EPIYA motifs. EPIYA region sequences of 14 isolates including reference strains (196A, 26695, ATCC43526, and P12) are shown (red). 9 isolates show the KDKGPE motif (blue) in front of the EPIYA-A motif [28].

To obtain further information on virulence factor characteristics, the CagA EPIYA regions of 14 representative strains were sequenced (Fig 3). This region consists of three different EPIYA motifs (A, B, and C), and it has been postulated that increased numbers of EPIYA-C motifs correlate with stronger pathology in the patient [27]. Nine of the 14 isolates sequenced here contained an ABC motif arrangement, the other five harbored either an AC motif (three cases), an ACC motif (one case) or an ABCC motif (one case). Conspicuously, a conserved KDKGPE motif was found upstream of the EPIYA-A motif in nine out of 14 sequences, however no correlation with pathology could be observed.

### 4.4 Characterized *H*. *pylori* isolates cluster with strains of the *hpAfrica1* population and have a characteristic deletion in the ICE*Hptfs4b* plasticity zone

We have previously shown that the integrating conjugative element ICE*Hptfs4b*, a plasticity zone which carries the duodenal ulcer promoting (*dupA*) gene and its associated T4SS that has been implicated in the formation of duodenal ulcers, is incomplete in several *H*. *pylori* isolates from Western Africa. In particular, one specific gap was observed when compared to non-African strains [22].

**Fig 4:**
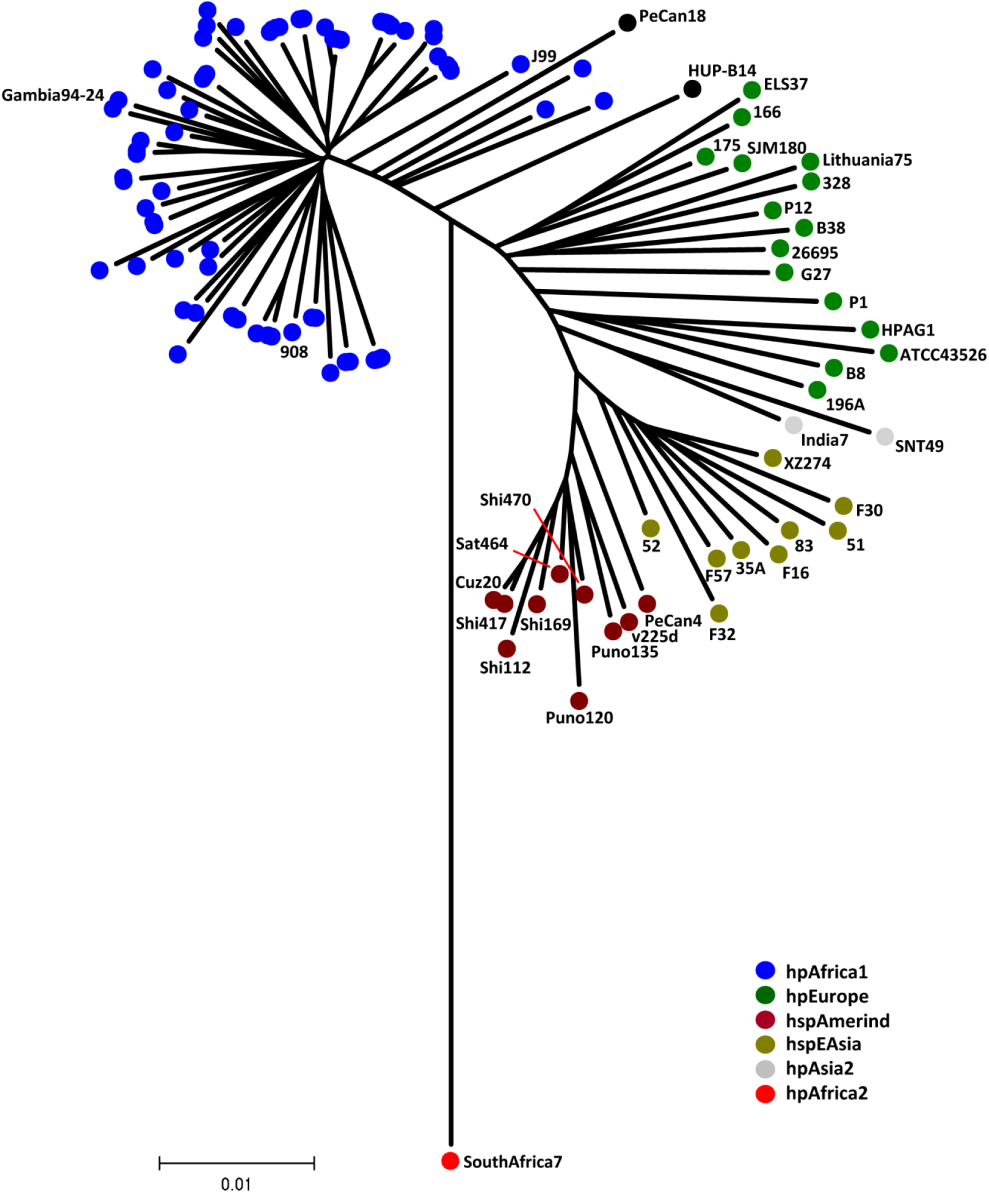
Phylogenetic tree of strains used in this study and representative strains from different populations. All strains used in this study are represented by blue dots, cluster with the hpAfrica1 (hspWAfrica) strains 908, Gambia 94–24, and J99. Strains from other populations are color-coded as indicated.

Utilizing PCR analysis, we observed a truncated right region of ICE*Hptfs4b* identical to the one previously described in 64 out of 65 strains analyzed, indicating that these strains do not carry a functional *dupA* T4SS (data not shown). This observation further suggests that most, if not all, West African strains carry this type of deletion. However, since it was observed that the chance of successfully isolating *H*. *pylori* was much higher from hospitals located close to Lagos, where the strain-isolation laboratory was located, one possibility was that the analyzed strains are simply very closely related. Thus, 78% of the 111 characterized strains originated in Lagos. To exclude that a generally low variance of the examined *H*. *pylori* isolates was the reason for the genotype similarities, an MLST analysis was performed. MLST sequence analysis indicated that all sequenced strains were *hpAfrica 1* (*hspWAfrica*) strains, which was reflected by clustering of the Nigerian isolates together with the reference strains 908 [29], Gambia 94–24 [30], and J99 [18] (Fig 4). This analysis also indicated that at least six patients were infected with multiple strains of *H*. *pylori*, as isolates originating from the same patient did not cluster within the phylogenetic tree (data not shown).

## 5 Discussion

The UBT is still considered to be the gold standard in diagnosing *H*. *pylori* infection, especially in developing countries where it is an often used and non-invasive test [31]. But we showed clearly that UBT-negative patients can also be infected with *H*. *pylori*, as we have successfully isolated *H*. *pylori* from five patients without positive UBT results. Since the UBT is, in general, a reliable method to diagnose the presence of *H*. *pylori*, the most likely cause of this is failure in either the technical performance of the test, or the assistance of the patients. Regardless of the reason, it remains one of the most appropriate methods as the alternatives are limited to: (i) biopsies, possible only in a few hospitals in Nigeria; (ii) analyzing stool samples, which is still a less reliable assay than the UBT [32]; or (iii) using the CLO test, which is less sensitive (as shown in the present study). Furthermore, even if the option to perform a gastroscopy and to isolate biopsies is given, it remains expensive and is not an option for every patient. Thus, the UBT remains the most reliable way to diagnose *H*. *pylori* infections.

As previously mentioned, the state of antibiotic resistance in Nigeria has been poorly studied. In the current study we demonstrated that 99.1% of isolates were resistant to metronidazole, with a significantly lower resistance rate to amoxicillin and clarithromycin, and a very low resistance rate to tetracycline. Reported rates of antibiotic resistance throughout Africa are predominantly higher (except for metronidazole) [33], with published rates of amoxicillin resistance between 46% and 83%, clarithromycin resistance between 28% and 45%, tetracycline resistance between 58% and 84% and metronidazole resistance ranging from 48% to 72%. Thus, when compared to other African countries, the resistance rates against amoxicillin, clarithromycin, and tetracycline are comparatively low in Nigeria, but for no other African country a metronidazole resistance rate of almost 100% has been reported. This might be explained by the widespread use of metronidazole in Nigeria. As metronidazole is effective not only against various bacteria but also against protozoa, it is very commonly used; mainly for the treatment of diarrheal diseases [34], gynecological infections [35] and menstrual symptoms [36]. In addition, 27.2% of the patients stated that they are taking antibiotics during an acute episode (mostly before seeing a medical doctor) and 20.1% indicated taking antibiotics regularly. Through many indications as well as the over-the-counter availability of antibiotics in Nigeria the intake of metronidazole as well as other antibiotics is uncontrolled and frequent, which is in turn due to differences in health care systems when compared to other countries. Officially a public health insurance system covers the primary health care of each Nigerian citizen, however this system, founded in 1999, is still in the implementation phase [37]. Because of this the private expenditure on health (in 2012) as a percentage of total expenditure on health was 68.9% in Nigeria, as compared to 23.7% for Germany in the same year. Furthermore, general government expenditure on health as a percentage of total government expenditure was 6.7% in Nigeria, as compared to 19.1% in Germany [38]. The outcome of these factors is that the majority of treatment costs need to be covered by the patients themselves, which often results in pre-emptive self-treatment by the patients.

A study performed in Germany in 2013 reported that in untreated patients *H*. *pylori* isolates showed a resistance rate of 36.2% to metronidazole and 20.9% to clarithromycin. By comparison, a significantly higher resistance was detected in pre-treated patients; resistance to metronidazole reached 69.7% while resistance to clarithromycin reached 73.7% [39]. However, since patients pre-treated with antibiotics were not excluded in the present study, and the percentage of pre-treated patients could not be determined unequivocally, a conclusion as to the significance of pre-treatments on antibiotic resistance could not be drawn.

Thus, the lack of detailed information about self-medication as well as the knowledge about uncontrolled and frequented intake of antibiotics in Nigeria might be responsible for the high resistance level of 99.1% against metronidazole.

The antibiotic resistance of patient isolates was analyzed by MIC test, furthermore the 23S rRNA of clarithromycin resistant isolates was sequenced and transformed into sensitive *H*. *pylori* strains. We clearly showed that other point mutations outside the 23S rRNA, or even other mechanisms have to be responsible for the observed clarithromycin resistance phenotype. There are several conceivable mechanisms which have been described in other species, such as efflux pumps [40], methylating enzymes [41], modulation of bacterial gene expression [42], or other mechanisms. Further characterization of these patient isolates may lead to the identification of novel, yet unknown mechanisms of clarithromycin resistance in African *H*. *pylori* strains.

In total, there were very few differences in the microbiological characteristics of the 111 analyzed isolates. Almost 100% of the isolates were CagA-positive and were able to translocate CagA. Considering *vacA*, all isolates were of the s1/m1 genotype.

Our results suggested an average pathology analyzed by gastroenterology reflected by two gastric cancer cases and 15.5% ulcer cases of all 577 patients (independent of a proven *H*. *pylori* infection). Earlier work suggested that *H*. *pylori* infection is associated with a 2-fold increased risk of developing gastric cancer, with cumulative incidence rates of 1–2% [43], and a 4-fold risk of developing ulcers (cumulative incidence rates 15–25%) [44]. The ulcer risk increases to 25-fold in cases where the *H*. *pylori* infection is located in the antrum [45]. Thus, compared to published data, Nigerian patients of the current study exhibited an average disease outcome. It should be noted that no ulcer was confirmed in histology. This is likely due to the fact that ulcer edges are usually presented as granulated tissue, which may be an ulcer or can alternatively indicate lesions in the mucosa. Discrepancies between gastroenterology and histology in the identification of features such as ulcers and cancer have been previously reported [46]. Considering EPIYA regions of *cagA*, we discovered that the majority of patients had a KDKGPE motif upstream of the EPIYA-A motif. This motif was previously observed in strains of different origin [47], however no further experiments regarding its function were performed. Whether this motif is unique to African strains and whether it may be linked to pathology in Nigerian patients, has to be clarified in further studies. Likewise, the aforementioned gap in the ICE*Hptfs4b* plasticity zone, which carries the duodenal ulcer promoting (*dupA*) gene, might be an interesting basis for functional correlation studies. In conclusion, our data support and extend the findings of previous studies and suggest that African strains from our study cause a normal pathology, but are generally *cagA*- and *vacA*-positive.

In summary, we present the results of a cross-sectional study of *H*. *pylori* infection within this patient cohort with a low infection rate. In addition, we found a uniformly high level of resistance to metronidazole, which might partially be related to the clustering of sample locations, but may also relate to differences in health care practice in Nigeria resulting in an uncontrolled drug intake. Furthermore, we show a uniform presence of the major virulence factors causing an average pathology.
